# Women’s sexually transmitted infections in primary care: General practitioners’ challenges and strategies – A qualitative study in Germany

**DOI:** 10.1080/13814788.2023.2190094

**Published:** 2023-04-03

**Authors:** Pauline Meurer, Christoph Heintze, Angela Schuster

**Affiliations:** Charité – Universitätsmedizin Berlin, corporate member of Freie Universität Berlin and Humboldt-Universität zu Berlin; Institute of General Practice, Berlin, Germany

**Keywords:** STI, women’s health, primary health care, general practice, Germany

## Abstract

**Background:**

The incidence of sexually transmitted infections (STI) is rising. Amongst women, STIs are often asymptomatic and thus likely underreported. STI care in Germany is fragmented. General Practitioners (GPs) could offer accessible care; however, to which extent GPs provide STI care and which challenges they face remains unclear.

**Objectives:**

To increase understanding of how GPs provide STI care for women in German high-incidence settings and to identify challenges and opportunities for improved care.

**Methods:**

Between 10/20 and 09/21, we contacted 75 practices using snowball and theoretical sampling. We conducted qualitative guide-assisted interviews with 19 GPs in their practices in Berlin, Germany. Data were analysed using thematic analysis with grounded theory components.

**Results:**

Responsibilities and financing of STI care services were unclear. Most GPs perceived specialised doctors to be responsible for STI care in women; however, many non-STI specialised doctors were the first point of contact for patients and felt responsible to help. (LBTQI) Women were perceived to have less access to care. Stigmatising perceptions of women with STI-related needs were common. Doctors immediately referred patients to other providers, offered STI care for selected cases, or routinely offered primary STI care. GPs’ referral strategies were often unsystematic. Those who offered primary STI care perceived patients’ need for STI care, showed open attitudes to sexual health, and had undergone further training on STI care.

**Conclusion:**

Training regarding STI care, remuneration, and referral pathways should be provided for GPs. Comprehensive STI care could be offered through the cooperation of GPs and specialists.

KEY MESSAGES

- STI care is hampered by unclear responsibilities, referral pathways and financial remuneration, by stereotypes and uncertainties regarding diagnostics.
- GPs feel responsible for helping; they either rely on referrals, provide care for selected cases, or provide comprehensive STI care on-site.
- GPs with appropriate training and open attitudes provide comprehensive STI care.

## Introduction

Rising incidences of sexually transmitted infections (STI) are reported in the US and Europe [1,2]. Between 2010 and 2017, the syphilis incidence rate rose by 153% in the UK and by 144% in Germany [3]. The number of cases is particularly high in urban settings; in Germany, Berlin is the most affected city [4].

The most common STI in England and Germany is chlamydia, with 9% and 5% of 15-24-year-old females testing positive in screenings in England and Germany, respectively [5,6]. Chlamydia is asymptomatic in up to 80% of women [7], and the German screening programme for pregnant persons and women aged 15–25 reached only 11% of the target group in 2015 [6]. In women, STIs can ascend through the cervix and into the upper genital tract and cause serious sequelae such as pelvic inflammatory disease and infertility [8,9].

Before the 1970s, sexual health care in Europe was mainly covered by non-governmental organisations (NGOs). As sexual health was integrated into regular health care, different providers took on the responsibility: general practitioners (GPs) in the UK and the Netherlands, community health centres in Sweden and Portugal, and gynaecologists working at the primary care level in Germany and Poland. Often, STI care is separated from family planning, and specialised care structures have emerged during the HIV crisis [10]. Most people living with HIV in Germany are followed-up in HIV-focussed practices, usually led by general practitioners or internal specialists with an additional qualification in infectious diseases. They provide both primary care and specialised care for HIV and STI.

As a result, sexual health care in Germany is fragmented and characterised by a multitude of actors. Many ambulant services are designed for specific groups, e.g. men who have sex with men (MSM) or sex workers. Health services are offered by medical care providers (gynaecology, urology, dermatology, general medicine, STI clinics) but also by the local health authorities and NGOs. The latter focus on populations who experience difficulties in accessing the regular health system, they offer STI testing at low or no cost but can’t always provide STI treatment [11]. Patients can freely consult GPs, specialists, or other providers according to their preferences. Specialised doctors can be consulted directly; referrals from the GP are not required.

The guidelines of the German STI Society recommend counselling and diagnostics for either symptomatic patients or according to so-called epidemiological criteria, which are based on behavioural risks or on belonging to a population considered at risk [12]. Guidelines provide orientation for care but are not legally binding. The remuneration of GPs in Germany is based on a quarterly lump sum per patient, independent of the complexity of provided service. Based on the number of patients, a laboratory budget is calculated. In specific occasions, including suspicion of notifiable STI, laboratory tests can be carried out without affecting the budget. Notifiable STIs must be reported to the Robert Koch-Institute, including syphilis, HIV and gonorrhoea with reduced sensitivities to certain antibiotics. Other STIs can also be tested budget neutrally if notifiable diseases are suspected; laboratory costs are then directly covered by German compulsory health insurance. Otherwise, costs up to 200 euros for STI/HIV testing are covered either via the doctor’s laboratory budget or out of pocket by the patients themselves. From the practices’ economic perspective, accounting advisors recommend billing every STI test in asymptomatic patients as an out-of-pocket service. Recommendations from a medical perspective are lacking, the German guideline does not comment on cost coverage [12].

Out-of-pocket billing seems common, especially among gynaecologists [13]; however, systematic data for out-of-pocket billing in Germany are lacking.

Care of women’s sexual health in Germany focuses on reproductive health. Women mainly address gynaecologists for this need [10], usually once a year as part of a preventive check-up. GPs are rarely consulted for gynaecological needs, although over 80% of women of all ages use GP care within one year and sexual health counselling is anchored in the General Practice residency training [14]. Thus, GPs could offer accessible care across age, gender, and cultural backgrounds and complement care offered by gynaecologists. However, in a survey among German GPs, 43% felt insufficiently trained to provide preventive counselling on STIs, and 36% reported referring more than half of the patients with STI-related needs to specialists or the local health authorities [15].

### Rationale

Rising STI incidence, fragmented care, and unclear financial remuneration may hamper STI control and universal access to sexual and reproductive health, as stated in the Sustainable Development Goals (SDG 3, Target 3.7) [16].

The German federal strategy for STI control (BIS 2030) aims for needs-based, cross-sectoral care provision [17]. GPs, as the first point of access to health care, take a central role in reaching those goals. However, while they perceive their competencies for STI care as insufficient [15], their self-perceived role and strategies in STI care provision in Germany have never been assessed qualitatively.

## Methods

Our research aims to provide a deeper understanding of how, and to which extent, GPs provide STI care for women, and which challenges GPs face. Our research design was guided by qualitative content analysis, using semi-structured, guide-assisted interviews. This allowed us to conduct interviews in the limited time available to GPs while leaving room for individual interpretations.

PM, a female medical doctoral student with training in social science methods and theory, conducted the interviews. The process was supervised by AS (post-Doc) and CH (Professor for Primary Care), medical researchers with experience in qualitative methodology. Both AS and PM are interested in sexual health and hold an intersectional feminist stance. We assumed that women are underserved concerning STIs.

We aimed to interview GPs likely to face sexual health needs regularly in their practice. The STI incidence is highest in Berlin and the metropolitan areas of Germany [6]. Therefore, we decided to focus on GPs in Berlin.

PM contacted participants in her role as a doctoral researcher by telephone or e-mail. The interview partners were informed in writing about the aim of the study, they did not meet the interviewer before the interview. The interviews were conducted in the doctors’ practice rooms, mostly after office hours. No other persons were present.

### Sample

The sample was developed iteratively. First, we contacted GPs who had participated in an introductory seminar on sexual health and then we contacted GP practices located in the vicinity of randomly selected geographical areas in the metropolitan area of Berlin, using Google Maps. Further, we contacted GPs recommended to us by snowball sampling. Theoretical criteria were then applied to extend the sample: We included GPs without an STI focus and GPs with an additional specialisation in infectious diseases and HIV. We ensured an equal distribution of gender. GPs from different areas within Berlin were included to account for different population needs and age structures at the district level. Younger doctors who were still training to become GPs were included, as we expected generational differences in the attitudes towards sexual health.

We contacted 75 practices; most did not respond to our inquiries and 10 practices or doctors refused to participate in the study, mostly due to a lack of time.

The sampling was stopped after interview 19, as saturation of information had been reached. Of 19 participating GPs, six worked in infectiology/HIV-focussed practices (coded as S01 to S06), and 13 in practices without an infectious disease focus (coded as GP01-GP13). Four of the doctors were still in further training. The gender ratio was balanced in all sub-groups; nine cis-women and 10 cis-men took part in the interviews. The age of the participants ranged from 32 to 63 years.

### Ethics

Ethical approval has been granted by the Ethics Commission of Charité – Universitätsmedizin Berlin, Ethical review number: EA1/188/20. All participants provided written consent. Interview documentation and transcription were pseudonymised, full names were not used. Names of the interview partners were not recorded. Identifying names that appeared in the interviews were paraphrased according to their function.

### Data collection

The interview guide was developed by AS and PM and pretested with one GP before conducting the interviews. Evaluation of findings and interviewing took place in parallel in an iterative process. Initial findings influenced the further course of the research, new questions were included in the guide, and the sample was adjusted. To account for doctors’ experience with medical cases, we integrated three case vignettes as narrative stimuli, these can be found in supplement 1.

The latest version of the guide can be found in supplement 2. All interviews were audio-digitally recorded and transcribed verbatim. Since GPs were severely limited in time availability, interpretations were not validated with participants (member-checking). Interviews were conducted between October 2020 and September 2021, with interviews paused during winter due to an increased workload secondary to COVID-19. Field notes were taken during and after the interviews. The interviews ranged in length between 22 and 68 min, with a mean of 47 min.

### Data processing and analysis

Transcription, coding, and analysis of the interviews were done using MAXQDA^®^ software on a password-protected institutional server. First, case summaries were written and themes for coding were identified. The coding process was carried out inductively on the material and deductively referring to existing literature. We structured the themes into four superordinate categories: structural conditions, attitudes, knowledge, and care strategies. Within the category of care strategies, both provided care and coordination and referral strategies were examined. The complete coding tree can be found in supplement 3.

We used thematic analysis with grounded theory components [18]. To extract and visualise the relationships between categories, we used a coding paradigm proposed by Strauss and Corbin and adapted by Flick [19] (supplement 4). Factors influencing care provision were identified through continuous comparisons between cases and within cases.

PM coded all interviews; AS and a doctoral student counter-coded six interviews. When different assessments of the material emerged, these were discussed, and a consensus was reached. All thematic categories were summarised in a case-based summary grid to achieve a higher level of abstraction without deviating from the material.

Doctors in HIV/STI-focussed practices usually specialise in infectious diseases and have a higher laboratory budget. Hence, we focussed on GPs without a contagious disease specialisation to analyse care provision. Two doctors in further training could not be assessed in terms of their care strategies. They had minimal work experience and mainly reflected the strategies of the practice owners. The analysis of care provision and their influencing factors thus focussed on a group of 11 GPs. All 19 interviews were used in the analysis of other categories.

Reflection on the contents and interpretation of data occurred within the research colloquia of the Institute of General Practice and the Institute of Medical Sociology.

## Results

### Structural conditions

Doctors who did not see STI care as their responsibility emphasised a lack of time and budget. However, the lack of time for holistic care or cooperation with other doctors was mentioned by many GPs. Several interview partners shared their struggle to cover STI diagnostics via the lump sum payment per patient’s health insurance. The expensive PCR test would conflict with the demand for cost-effectiveness in practice.

***S03:*** *On the one hand, we are told to provide a good medical service, but on the other hand we are also supposed to work cost-effectively, how is that supposed to work? If a smear test costs 180 euros […]. (S03, male, aged 39 years)*

The responsibility for STI care was described as unclear and allocated to different providers:

***GP03:*** *[…] Where can I send someone? I find that difficult. You always build your own structure piece by piece, but no coordinated care pathways exist. I say I have someone here, they are responsible for it, and then I send them (the patients) there, and then they are cared for. That doesn’t exist. (GP03, male, 63 years)*

Most GPs felt professionally responsible when patients approached them as a first point of contact but did not feel formally in charge of women’s STI care. However, some doctors did feel formally responsible for men’s STI care.

Women’s STI care responsibilities were perceived to lie with different healthcare providers. Some practices with an infectiology/HIV-focus did not provide care for women. While some interview partners stated that the women’s gynaecologist would care for sexual health care needs, other interviewees said that women with sexual problems do not address gynaecologists for sexual health.

***S02:*** *No, that’s a bit of a perception, you kind of think that women can always go to the gynaecologist, but […] it’s actually becoming increasingly clear to us that women do go to the gynaecologist regularly, but not with sexual problems. (S02, male, 51 years)*

Interviewed doctors considered women to have less access to STI care services, which were often primarily developed for men.

***S06:*** *I think that just like in addiction medicine, also in HIV medicine, infectious diseases, and STI, the programmes are much more tailored to men. […]. (S06, female, 53 years)*

Access to care was described as particularly decremental for transgender patients or patients with a non-heteronormative sexual orientation:

***GP04:*** *It is much more difficult for transgender people in any case, where there is much less openness, overall, I would also say among medical staff. […] I think there are probably the most deficits in care, and for women who have sex with women, bisexuals, transgender people, I think there are fewer direct offers. (GP04, female, 50 years)*

### Knowledge

GPs reported uncertainties regarding STI diagnostic workups and therapy. Many were unaware of the possibility of deducting the extra lab expenses from their laboratory budget.

Some GPs stated having expertise in STI care but reported knowledge and research gaps for STI care in women and particularly trans women. Specialised gynaecological equipment, such as a gynaecological chair, was often deemed necessary to provide care. Many GPs considered gynaecological examination a particular challenge.

Some doctors were unaware of asymptomatic infections in women and explicitly described assessing the risk based on symptoms.

***GP05:*** *I would tell her it’s her private pleasure, if she has no symptoms, it’s just a preventive measure, and the health insurance does not pay for preventive measures at the moment. (GP05, female, 52 years)*

### Attitudes

Attitudes towards sexuality and STIs varied among the interview partners. Some doctors expressed understanding and were aware of their patients’ potential stigmatisation and marginalisation. Other GPs showed stigmatising attitudes and associated STIs with prostitution, clubs, alcohol abuse, or belonging to a certain (low) social class, and blamed patients for contracting an STI.

***GP01:*** *[…] it is often really a risk for humanity, such women, when they practice (sex) like that. (GP01, male, 61 years)*

Classical gender roles, such as the consideration that a woman would use contraception responsibly, were formulated.

***GP05:*** *[…] because she (the woman) is usually a bit more sensible, I hope she says: ‘Hey, you, remember, do you have the condoms with you?’ (GP05, female, 52 years)*

While the described challenges regarding knowledge, attitudes, and practices affected all genders, female patients were defined as over-proportionally affected (Table 1).

**Table 1.** Summary of challenges regarding STI care: analysed results.

	Structural	Knowledge gaps	Attitudes
Challenges in STI care	Lack of time Limited financial remuneration Unclear responsibilities	Uncertainties with diagnostics and therapy Low awareness of asymptomatic infections Uncertainty regarding recompensation of lab expenses	Stigma and shaming
Additional Challenges in STI care for (LBTQI) women	Less access to STI services for women and LBTQI persons Responsibilities particularly unclear due to gynaecological involvement	Assumption of needing a gynaecological chair for taking a smear Additional knowledge gaps in trans women STI care	Gender stereotypes Lack of openness for LBTQI persons

### Care strategies

#### Types of care provision

We identified three different STI care provision types. Some doctors immediately referred patients to specialists, some offered STI care for selected cases, and some routinely offered primary STI care (see Table 2). While referring patients can be interpreted as primary care, we understand primary STI care as on-site care: Counselling, diagnostics, and therapy are provided at least initially in the GP practice.

**Table 2.** Types of care provision: analysed results^a^ and illustrative quotes.

	Type of care provision		
	Referral	Selective care	Primary care
Care provision	Care exclusively for urinary tract infections, STI care or counselling are not offered. Patients are referred to specialists.	Partial or varying provision of care - in exceptional cases, care is provided beyond basic diagnostics, e.g. initial treatment if no acute appointment with a specialist is available.	Routinely take a sexual history, and offer counselling. Provide for diagnostics and therapy of STI, at least initially, also for women.
Responsibility for STI care	Formal responsibility lies with specialists or patients themselves. No sense of professional responsibility for STI care	Formal responsibility lies with specialists. Professional responsibility to help patients in some way	Formal responsibility lies with specialists or with the local health authorities. Professional responsibility to provide primary care
Illustrative quote	GP06: […] I would think, that is your problem. […] You can discuss that with the gynaecologist, and I don’t do the tests either. Because that’s simply not my area. (GP06, female, 61 years)	GP03: And in such cases I say ok, before there is no treatment […], I'd better start and do it. (GP03, male, 63 years)	GP10: […] That’s why we’re primary care providers, so people can get their tests. (GP10, female, 45 years)

^a^Case level and across-case analysis using a grounded theory-based coding paradigm developed by Strauss and Corbin and adapted by Flick [19], for coding paradigm see supplement 4.

#### Factors influencing care provision

The doctors’ perception of STI care needs to be varied according to their attitudes and training. Table 3 summarises the main variations according to the type of care provided.

**Table 3.** Factors influencing care provision: analysed results^a^ and illustrative quotes.

	Type of care provision		
	Referral	Selective care	Primary care
Perceived need	STIs are rarely or never an issue	STIs are rarely an issue, mostly men present with STI needs, women are more likely to seek care in a gynaecology clinic	Perceived regular need for STI care
Relevant education and training / knowledge	No education nor training for STI care	Mostly no education nor training, partly anecdotal experience	Relevant training, desire for more information
Attitude towards sexual health	Stereotyping perceptions; emphasis on the potential danger. Use of stigmatising language	Varying, some showing stereotyping perceptions, some being very open	STI as a normal occurrence, open attitude towards sexuality
Illustrative quote	GP05: There are simply a lot of idiots who still do it without a condom, who need the thrill. (GP05, female, 52 years)	GP01: […] Because such cases are sometimes, let’s say, people who come from the lowest class, who are not too educated […]. (GP01, male, 61 years) GP07: I think it’s really cool that she’s so open about it and that she also says she wants to be tested. (GP07, female, 54 years)	GP10: […] I think, yes, sure it can happen, it’s good that she’s here, it must be treated, no drama. (GP10, female, 45 years)

^a^Factors influencing care provision were identified through continuous comparisons between cases and within cases.

The main distinction between doctors offering primary STI care and the other two groups was an open attitude towards sexual health, relevant STI training, and the perceived need of patients for STI care. It seems plausible that knowledge and an open attitude are prerequisites for the perception of need.

#### Coordination and referral strategies

Most GPs relied heavily on referrals to specialists. While doctors were uncertain about how much STI care gynaecologists provide, they referred patients there anyway.

Doctors who also provided primary STI care referred to specialists they knew and who provided STI care, usually dermatologists or infectious diseases practices. These doctors explicitly wished for better cooperation with gynaecologists.

Although public sexual health centres are comparatively present in Berlin, these local health authorities were rarely mentioned by GPs. Most GPs were not aware of the health authorities’ role and responsibility.

## Discussion

### Main findings

STI care was seen as an additional task with unclear responsibilities, financial remuneration, and uncertainties regarding diagnostic workup. Stigmatisation of patients with STI-related needs and gender role-specific preconceptions were common.

The primary responsibility was almost always seen in the hands of specialists but GPs’ professional responsibility as the first contact person was very present. The responsibility for women’s STI care was particularly unclear since gynaecologists are perceived as responsible for women’s sexual health. Nevertheless, women were perceived to have difficulties accessing STI care, and additional access barriers for queer women were mentioned. GPs feel left alone with their patients and with unclear responsibilities and cost coverage for STI diagnostics.

Yet structural challenges were not decisive for the care strategies. Doctors with appropriate training and an open attitude also saw a need, took on STI care, and offered targeted referrals.

### Strengths and limitations

We interviewed GPs in Berlin; the results are not directly transferable to other, more rural, regions in Germany. However, since the STI burden is mainly concentrated in urban areas [6], we think that the identified needs and challenges are transferrable to most high-incidence settings in Germany. The combination of structural difficulties, namely lack of time, unclear responsibility, and unclear financial remuneration, is specific to the German healthcare system, but similar challenges apply to other countries.

We could not discuss results with the participants and could only interview a relatively small number of GPs. However, analysing a small and homogeneous group is also an advantage, allowing for more detailed and specific comparisons. The additional perspective of GPs specialised in HIV and infectious diseases broadened the results. The interview approach using case vignettes enabled us to identify concrete problems in day-to-day practice.

In our sample, neither the age nor gender of doctors, nor the location and population structure of the practice, were decisive for the type of care provision. However, quantitative surveys are needed to investigate the influence of these factors further. Though no focus of our interviews, GPs reported reduced access to STI care for women in racialised and LBTQI communities. Further research in this regard is needed.

### Comparison with existing literature

STI care provision from the GPs’ perspective has not yet been described qualitatively in Germany. Through the analysis of the three types of STI care identified in this study, the variation of care strategies among GPs was illustrated and the significance of personal attitudes and knowledge of the doctors about STIs became apparent.

German physicians tend to initiate diagnostics when symptoms are present [20]. Accordingly, several interview partners offered only symptom-based STI counselling and testing, suggesting low awareness of asymptomatic infections. Depending on the pathogen and the site of infection, up to 90% of STI infections are considered asymptomatic [12].

Some of the interviewed doctors perceived women as more reasonable, reproducing presumptions related to gender roles and potentially leading to underestimating risks. Risk underestimation is reflected in multiple epidemiological studies leading to late diagnosis and treatment with worse outcomes. Epidemiological data show that 19% of heterosexual women were diagnosed with syphilis more than a year after infection, compared to 10% of heterosexual men [4]. Similarly, women are more likely to have a late HIV diagnosis despite showing indicators of HIV infection [21].

Risk assessment for STIs is in a field of tension: existing statistical differences between STI incidence in different groups (MSM; sex workers) need to be considered, yet there is a danger of bias and a shifted risk perception. Implicit bias is reinforced by uncertainties and lack of time for person-centred care [22]. Biases could be reduced with specific strategies: consciously taking the patient’s perspective and focussing on the patient’s individual information instead of thinking in social categories [23].

Some doctors saw the responsibility in the patients themselves, resulting in a lack of service provision. This attitude represents a sense of culpability, which increases stigma [24]. For potentially stigmatising diseases, clear policies and responsibilities would be essential [22].

Multiple discrimination, e.g. in women from racialised communities, sex workers, and LBTQI women, needs to be considered. In our interviews, little knowledge and a lack of openness among medical staff for the care of trans women were described. Interviews with healthcare providers in the UK identified similar barriers to trans health care: lack of guidelines, insufficient education among practitioners, and negative attitudes [25].

In Germany, GPs can choose areas of focus, resulting in considerable differences in care delivery and assumption of responsibility; while some of the doctors in our interviews never took a sexual history, others offered comprehensive STI care.

As a result of the challenges in STI care and in line with quantitative surveys [15], many doctors reported referring patients to specialists or, less frequently, to the local health authorities. Likely, services offered by specialists and local health authorities vary considerably and not all services do provide comprehensive STI care [26].

Anchoring sexual health with GPs would increase access to needed care, especially for populations with lower socioeconomic status and patients in rural areas [14]. Further costs for specialised care could be reduced. Sexual health care largely consists of preventive and counselling measures, which fall within the remit of general practice [10]. There are strong calls for more preventative medicine, a more robust public health focus and more primary care in Germany. A primary physician system with a gatekeeper function and a binding catalogue of services for GPs, as realised in other European countries, has been proposed.

### Implications for practice

Strategies for improved STI care should include targeted information, rising awareness with GPs, and regular training.

Communication of efficient referral pathways could contribute to improved care since they can easily be implemented by GPs with no specific interest in STIs. Information on financial remuneration might support doctors who offer care for selected cases in broadening their care. Implicit bias education could further improve the care of doctors who are already open to sexual health.

Neither the German federal strategy for STI control (BIS 2030) nor the clinical guidelines address GPs [12,17]. Specifically addressing GPs, as realised in the British STI guideline [27], could increase the sense of self-perceived responsibility.

The BIS 2030 strategy states that accessible counselling and testing services are not available everywhere. Integrated prevention, testing, and care services are to be further developed and the cross-sectoral networking of actors is to be promoted [17]. While GPs in Germany could take on a stronger role as a point of contact for the general population, specialised actors could complement care provision. Integrated STI care with transparent referral pathways could enhance the involvement of GPs and improve access to care.

## Conclusion

Unclear diagnostic workups, referral pathways, as well as financing of STI care are challenges for GPs. GPs use different strategies to navigate these challenges: relying heavily on referrals, providing STI care for selected cases, or providing primary STI care.

To reach different providers, targeted strategies are proposed: efficient referral pathways could be implemented by GPs with no specific interest in STIs, continued education and information on financial remuneration could enable doctors to broaden their care and implicit bias education could further improve the care of doctors already providing STI care.

## Supplementary Material

Supplemental Material

## Data Availability

The complete codebook with code definitions, anchor examples and pseudonymised interview material can be shared on request.
