# Supplementary material for: Women’s sexually transmitted infections in primary care: General practitioners’ challenges and strategies – A qualitative study in Germany
Source: Eur J Gen Pract. 2023 Apr 3;29(1):2190094. doi: 10.1080/13814788.2023.2190094 (PMC10071968; doi:10.1080/13814788.2023.2190094)
Supplement: Supplemental Material [file IGEN_A_2190094_SM5606.docx]

[Supplement 1: Case vignettes 2](#_Toc129858818)

[Supplement 2: Interview guide 3](#_Toc129858819)

[Supplement 3: Coding tree 9](#_Toc129858820)

[Supplement 4: Coding paradigm 10](#_Toc129858821)

#

# Supplement 1: Case vignettes

| Case 1 - Mrs West 44-year-old Mrs West has already visited your practice several times. Today she complains of pain when urinating, which has been present since yesterday, as well as increased discharge from the vagina. She knows the pain from previous bladder infections, but she has not had a bladder infection for a long time and the discharge is new. She would like a certificate of sick leave and something to relieve the pain. |
| --- |
| Case 2 - Mrs Leine Mrs. Leine is a 23-year-old patient known to you. She comes to you because a man with whom she had sexual intercourse a fortnight ago called her yesterday - he had been to the health department and had been diagnosed with syphilis. Mrs Leine is very worried and has many questions. She wants to know if syphilis is bad, if she could have contracted it, and what happens now. She asks whether she is in the right place here with you at all, or whether she would be better off going to the health department. |
| Case 3 - Mrs Biancharelli Mrs Biancharelli is a 32-year-old patient who comes to your practice. She would like to make sure that she does not have any STIs. Now and then she has had unprotected sexual intercourse. In the last few years, she has been single and has had sex with different partners on a regular basis. She has no symptoms, but has read that many sexually transmitted diseases can be completely inconspicuous. Friends had told her that a test for STIs would cost a lot of money - she is on a low income and cannot afford it. |

# Supplement 2: Interview guide

## Study project Sexually transmitted infections and their prevention in women in primary care

Introductory remarks

- Information material sent in advance; "have you had a chance to read through it?"
- Review important points together - "In this study project, we are interested in the role of GPs in the care of sexually transmitted infections in women. Since there has not been much research on this topic in Germany so far, we are conducting interviews with GPs like you as experts to discuss topics and problems openly. We are particularly interested in your personal opinions and experiences in practice. We would also like to be able to talk about concrete situations by presenting short case vignettes and discussing them with you - it is not about testing knowledge, but rather about everyday practice and the care situation as you experience it as a GP.“

*Note: for a better overview, the case studies have been kept separate. They are listed in supplement 1.*

- The interview will last approximately one hour and will be recorded. The data will be analysed and possibly published, but it will not be possible to draw any conclusions about the person.
- Show data protection sheet with contact details and point out the right of withdrawal; signatures; information and a version of the consent form remain with the participant.
- Note code on memo sheet
- Questions do not have to be answered, interview can be interrupted at any time
- Short factual questionnaire at the beginning

| Topic | Narrative impulse / stimulus | Memo Asking if not addressed by interview partner | Maintaining the flow of conversation / steering questions |
| --- | --- | --- | --- |
| A – Care provision | Non-specialist practices:  Perhaps you could start by telling me a bit about your career - why did you choose to work as a GP?  Imagine I would be in your practice as an intern for 4 weeks. In your experience - when would the issue of sexually transmitted diseases normally come up?  In the case of specialised practices / additional training or similar - ask them as an introduction: Why did you decide to work in this area / offer this type of care?  Can you tell me about the last patient who came to your practice with a concern about sexually transmitted diseases?  Do you feel the situation due to Corona affects the care of patients with other concerns - e.g. STI?  / Do you feel that fewer patients than usual come with such concerns? | - How often do you have such or similar cases in your practice? - What other STI-related reasons do women typically come to your practice with? | Can you describe the case / the process to me in more detail?  What was it like for you? |

|  | I would now like to present a first case vignette - as discussed, the case vignettes are not about testing knowledge, but quite pragmatically about everyday life in the practice, about which you as a GP know best.  The first case is about a patient who comes to your practice with specific symptoms and your basic approach to dealing with them. (Hand over printout and read aloud)  Can you describe how you have dealt with such cases in your practice so far? | - If necessary, specify the case: Sexual contact after a Tinder date, unprotected. - How often do you have such or similar cases in your practice? - What do you look for when taking the history of this patient? - What diagnostics do you offer this patient? What diagnostics do you generally offer in your practice in the area of STI? - What therapy would you initiate? Which STIs do you treat in your practice? - To what extent would you discuss further prevention options with the patient? / Would you discuss further points with the patient? | And how would you proceed?  I would be interested in understanding more about...  Today we are talking explicitly about sexually transmitted diseases. Outside of such a context, it is common to prescribe antibiotics for suspected UTI without taking a swab. Thinking about your everyday practice - would you normally include STIs in suspected UTIs?  If yes, when? Always? Only in certain constellations? |
| --- | --- | --- | --- |

| Flexible interim questions | In surveys on the topic of STI among GPs, colleagues sometimes say that they do not feel comfortable talking to patients. Are there any occasions for counselling that make you feel uncomfortable? Which ones?  If necessary, follow up - are there also occasions for counselling with women that make you uncomfortable?  If not, why not?    Were there any patients or situations that particularly stuck in your memory?  Why do you think this particular patient was so memorable? | In other health systems, for example in the Netherlands, GPs are the first point of contact for many sexual health issues. What do you think about this - in comparison to our more gynaecologically-oriented system?  There are specialised practices for STI and HIV, especially in Berlin. What do you think of this system?  Who are the contact persons for non-risk groups?  Are there certain patients with whom it is easier or more difficult for you to react empathetically?  Where would you send people from your personal environment for an STI test? | What sources of information do you use on the topic of STI?  Would you say you have a personal interest in STIs and their care?  How would you describe your own training regarding sexual health? |
| --- | --- | --- | --- |

| B Networking / Cooperation | We have now talked in detail about patients with symptoms, which was very informative. I would now like to describe a new case vignette, this one is about a concrete risk constellation and the networking between different care providers. | - How often do you have such or similar cases in your practice? - In which situations do you refer patients to other providers / facilities? - To whom do you refer patients? - What role does the health department play in the care? What role do counselling centres play? - To what extent do you exchange information with other providers? - In your opinion, who is most responsible for the care of the patients? - Do you follow the therapeutic progress of the referred patients? | You have already mentioned x and y as other providers. Do you also sometimes refer to z?  Can you describe in more detail how the cooperation looks like?  Do I understand correctly that you see x as more responsible than y? |
| --- | --- | --- | --- |

| C- Risk assessment | In the third and last case vignette, we are interested in the availability of preventive services, this time without a specific trigger. | - How often do you have such or similar cases in your practice? - What do you ask to assess the patient's risk? - How high or low do you assess the risk in this case? - What would you offer the patient in this situation? (What will be tested?) - What is the basis of your decision or assessment? - What do you understand by epidemiological risks? What role does this play in your daily practice / with your patients? - How do you charge for STI diagnostics? | Do I understand you correctly that you pay particular attention to which group a patient belongs to? |
| --- | --- | --- | --- |

| D - Individual and public health | The infection rates of various sexually transmitted diseases have increased in Germany in recent years. In your view, what could be strategies to reduce the infection rate? | - Do you know the Public Health Campaigns / the “Liebesleben” Campaign of the BzgA? If so, what do you think of the campaign? - What effect do you think such campaigns have? - Who do you think is most responsible for introducing measures to reduce the number of infections? - In your opinion, to what extent should GPs contribute to reducing the number of infections? | Did you think that the campaign had a concrete impact on people's individual behaviour?  Do I understand you correctly that you think the public health service is more responsible for reducing infection rates than GPs or other specialist groups? |
| --- | --- | --- | --- |
| E – Challenges and opportunities | We now arrive at the last question. If you could wish for something - what should change in your view to improve care for sexually transmitted diseases? | - What hinders the optimal care for female patients in your daily practice? - What do you think limits optimal cooperation with other actors? - Do you have any suggestions on what could be improved? | Can you perhaps describe an example of this for me again? |
|  | Are there points that are important to you that we have not yet addressed? | Check again if everything has been addressed |  |

# Supplement 3: Coding tree

| 1 Sense of responsibility | 4 Knowledge – Focus areas |
| --- | --- |
| 2 Conditions for action – structural | 4.1 Knowledge, sources of information |
| 2.1 Structure and care situation | 4.2 STI Education and Training |
| 2.2 Perception of doctors and professional policy | 4.3 Training, Career |
| 2.3 Perception of other doctors | 4.4 Focus areas |
| 2.4 Occasions, utilisation, patient clientele | 5 Care strategies |
| 2.5 Urban-rural differences | 5.1 Sexual history and risk assessment |
| 2.6 Billing options / Payment | 5.2 Diagnostics and Therapy |
| 2.7 Equipment / time | 5.3 Public Health / Prevention in practice |
| 2.8 Data protection | 5.4 Counselling |
| 3 Attitudes | 5.5 Billing strategies |
| 3.1 Personal attitudes – sexuality and risk | 5.6 Coordination and Cooperation with doctors |
| 3.2 GP-role / self-perception | 5.7 Referral doctors |
| 3.3 Patients – needs, competences | 5.8 Cross-sectoral referral and cooperation |
| 3.4 Patient responsibility – contraception | 5.9 Referral and cooperation with hospitals |
| 3.5 Uncomfortable occasions |  |
| 3.6 Preferences for patients |  |
| 3.7 Anecdotes |  |
| 3.8 Public Health Initiatives |  |
| 3.9 Societal attitude – sexuality and STI |  |

# Supplement 4: Coding paradigm

1. Coding paradigm as proposed by Strauss and Corbin, source: Flick 2018 [19]:

Context and Intervening Conditions

Causal Conditions

Consequences

Action strategies

1. Adapted coding paradigm:

Attitudes

Structural Conditions

STI-care provision

Knowledge
